# SubMDTA: drug target affinity prediction based on substructure extraction and multi-scale features

**DOI:** 10.1186/s12859-023-05460-4

**Published:** 2023-09-07

**Authors:** Shourun Pan, Leiming Xia, Lei Xu, Zhen Li

**Affiliations:** College of Computer Science and Technology, Qingdao University, Qingdao, China

**Keywords:** Drug–target binding affinity, Self-supervised learning, Mutual information, Multi-scale features

## Abstract

**Background:**

Drug–target affinity (DTA) prediction is a critical step in the field of drug discovery. In recent years, deep learning-based methods have emerged for DTA prediction. In order to solve the problem of fusion of substructure information of drug molecular graphs and utilize multi-scale information of protein, a self-supervised pre-training model based on substructure extraction and multi-scale features is proposed in this paper.

**Results:**

For drug molecules, the model obtains substructure information through the method of probability matrix, and the contrastive learning method is implemented on the graph-level representation and subgraph-level representation to pre-train the graph encoder for downstream tasks. For targets, a BiLSTM method that integrates multi-scale features is used to capture long-distance relationships in the amino acid sequence. The experimental results showed that our model achieved better performance for DTA prediction.

**Conclusions:**

The proposed model improves the performance of the DTA prediction, which provides a novel strategy based on substructure extraction and multi-scale features.

## Introduction

Drug development is a complex progress involving long research cycles, high costs, and low success rates, which could take several decades and 400–900 million dollars for a new drug from screening small molecules to market approval [1]. In the past few years, the information technology has been widely applied in computer-aided drug design (CADD) methods to accelerate the speed of drug development [2]. The prediction of drug–target binding affinity (DTA) is an important step in drug discovery, which provides information on the strength of interaction between drug molecules and target proteins. Therefore, the development of efficient and accurate algorithm of DTA prediction is of great significance in CADD.

Early computer virtual screening mainly focused on two types of methods: molecular docking [3–5] and ligand-based similarity [6, 7]. The molecular docking technique utilized the three-dimensional structure of protein targets and drug molecules, and the affinity can be predicted by simulating the docking process of proteins and molecules [8, 9]. However, the acquisition of three-dimensional structures is difficult, and large-scale molecular docking process is time-consuming. In contrast to molecular docking, ligand-based methods do not rely on the three-dimensional structure of molecules, which predict DTA by comparing new ligands with known ligands. However, when the number of known ligands is insufficient, the ability of ligand-based approach is limited. In response to these challenges, machine learning methods for DTA prediction [10–12] have been gradually introduced in the virtual screening and improved the performance of DTA prediction. Wang et al. [10] treated the interaction between drugs and targets as a binary classification problem. After extracting chemical descriptors of drugs and protein sequence information, an SVM model was used for prediction. KronRLS [11] used PubChem structure clustering tool [13] and Smith Waterman algorithm [14] to obtain similarity matrices for drugs and proteins, and the Kronecker product of similarity matrices was used to define similarity scores for drug–target pairs. To alleviate the limitation of linear dependence in KronRLS, SimBoost [12] constructed a drug–target similarity network and established a gradient boosting regression tree model for prediction. However, these machine learning methods rely on carefully designed handcrafted features, and the selection of these features depends on specific domain knowledge and experience [15] As deep learning (DL) methods have demonstrated superior learning capabilities over traditional machine learning methods in multiple fields, they have gradually been applied to solve problems in bioinformatics, including the DTA prediction [16–22]. DeepDTA [16] used protein sequence and molecular sequence information in two separate CNN networks. The output feature vectors were concatenated and fed into three fully connected layers to predict binding affinity. DeepCDA [17] combined CNN and LSTM to encode protein sequence and molecular sequence and proposed a bidirectional attention mechanism to predict DTA. FusionDTA [18] replaced the coarse pooling method with a novel multi-head linear attention mechanism to aggregate global information to address the issue of information loss. Additionally, the knowledge distillation was applied to transfer learnable information from a teacher model to a student model to solve the problem of parameter redundancy. As molecule could be represented as a graph, in which chemical atoms and bonds can be represented by nodes and edges. With the rapid development of graph neural networks (GNN), researchers have applied GNN models to DTA prediction. GraphDTA [19] used the topological structure information of molecular graphs and different GNN models for drug representation, while CNN was used to learn protein representation which is similar with DeepDTA. DGraphDTA [20] constructed protein graphs based on protein contact maps for the first time, then the GNN was used to predict DTA through molecular graphs and protein graphs. MGraphDTA [21] constructed a super-deep GNN with 27 graph convolution layers by introducing dense connections to capture both local and global structures of molecules. These methods indicate that deep learning networks can better capture the features of drugs and proteins. Due to the high cost and time consumption of laboratory experiments, the size of training dataset for drug discovery is limited, which may cause overfitting problems for machine learning methods and affect the generalization of learned features. Self-supervised learning can use unlabeled data for pre-training and transfer the learned model to downstream tasks, which can alleviate the requirement for labeled data. There are also self-supervised learning methods used in drug discovery [23]. InfoGraph [24] maximized the mutual information between graph embedding and substructure embedding at different scales to learn graph representations. MPG [25] compared two half-graphs and distinguished whether they come from the same source as a self-supervised learning strategy. GROVER [26] proposed two pre-training tasks: for the node/edge level task, it randomly masked a local subgraph of the target node/edge and predicted the contextual property; for the graph level task, it extracted the semantic motifs existing in molecular graphs (such as functional groups) and predicted whether these motifs existed for a molecule. However, most existing research integrated all structural features and node attributes of the graph to provide an overview of the graph, ignoring more fine-grained substructure semantics. Proteins are macromolecules composed of amino acids. There are 22 amino acids that make up an organism, which are represented by 22 letters and can be naturally represented as a sequence of letters. Sequence-based DL models can effectively consider the contextual relationships of the sequences. MATT-DTI [27] utilized three convolutional layers as the feature extractor, followed by a max pooling layer. A multi-head attention block was built to model the similarity of drug–target pairs as the interaction information for DTA prediction. TransformerCPI [28] used a one-dimensional convolutional gated convolutional network and gated linear unit instead of the self-attention layer in the Transformer encoder. However, current studies focus only on the single scale of protein sequences, and traditional sequence-based approaches process the whole sequence at once may lead to the loss of local information and neglect multi-scale features of proteins, so how to combine multi-scale information to improve the robust of protein representation is also an open issue. In order to overcome the limitations of existing methods, we propose a novel framework, SubMDTA, a drug target affinity prediction method based on substructure extraction and multi-scale features. For molecules, inspired by Wang et al. [29], a self-supervised learning method based on molecular substructure is proposed for molecular representation. During the pre-training phase, subgraphs are generated to obtain substructure information, and subgraphs are replaced according to their similarity relationships to generate reconstructed graphs. We simultaneously maximize the mutual information between the subgraph and the original graph, as well as between the reconstructed graph and the original graph, to improve the correlation between subgraph-level and graph-level representations. After pre-training, the trained model is fine-tuned in downstream tasks. For proteins, a BiLSTM method that integrates multi-scale information based on n-gram method is proposed for feature extraction. Finally, the drug and protein features are concatenated and fed into a Multilayer Perceptron (MLP) for DTA prediction. We compared our proposed method with several state-of-the-art methods and the experimental results demonstrate that our method significantly outperforms other methods on the Davis [30] and KIBA [31] datasets.

## Materials and methods

**Fig. 1:**
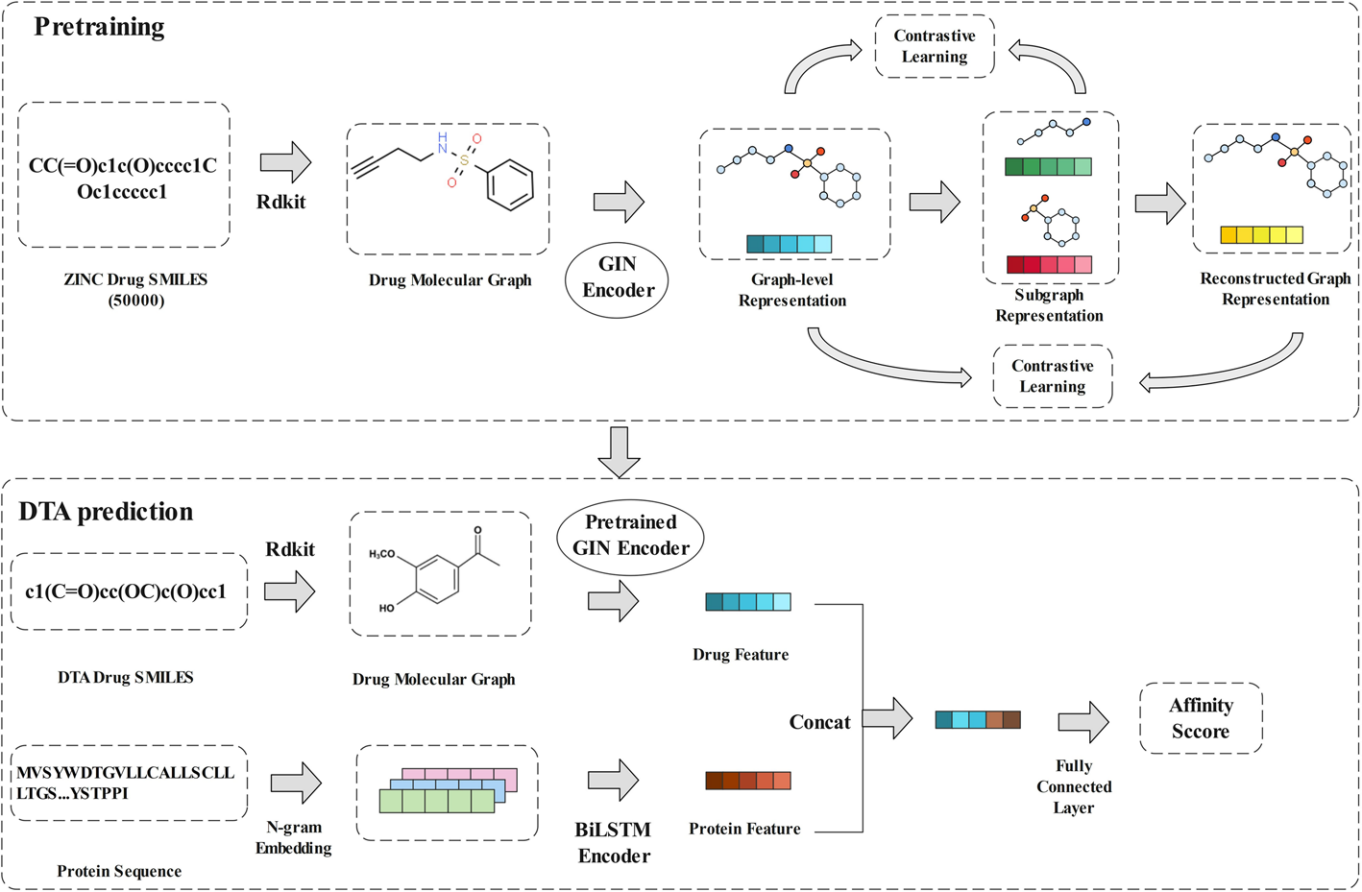
Overview of SubMDTA

The SubMDTA performs DTA prediction by integrating structural information of drug molecules and sequence features of targets, and the general architecture of SubMDTA is shown in Fig. 1. It consists of a pre-training part and a DTA prediction part. In the pre-training part, the drug SMILES (Simplified Molecular Input Line Entry System) [32] strings in the pre-training dataset are first converted into molecular graphs, followed by encoding the graph representations using the GIN [33] network. Then the substructural and reconstruct graphs are extracted. After obtaining two types of features, the mutual information between them and the original graph are maximized. The DTA prediction part uses the trained GIN encoder for molecular representation. For protein sequences, they are firstly embedded by n-gram coding, and fed into BiLSTM to obtain their representations. Finally, the drug representation and the protein representation are concatenated and fed into the fully connected layer to predict the binding affinity.

### Datasets

The Davis and KIBA datasets were used to evaluate the performance of the proposed model. The Davis dataset was obtained by selecting certain kinase proteins and their corresponding inhibitors, with binding affinity represented by the dissociation constant $K_d$, and affinity was processed using Eq. 1. It contains 442 proteins, 68 drugs, and 30,056 drug–target interactions. The average length of the drug SMILES strings is 64, and the average length of the protein sequences is 788. The KIBA dataset includes combined kinase inhibitor biological activities from various sources, such as inhibition constant ($K_i$), dissociation constant ($K_d$), or the half-maximal inhibitory concentration ($IC_{50}$), and predicts biological activity using the KIBA score. It consists of 229 proteins, 2111 drugs, and 118,254 drug–target interactions. The average length of the drug SMILES strings is 58, and the average length of the protein sequences is 728, the detail information of these two datasets is shown in Table 1.

1 $$\begin{aligned} pK_{d} = - {\log _{10}\frac{K_{d}}{10^{9}}} \end{aligned}$$

**Table 1:** Summary of the benchmark datasets

Dataset	Proteins	Compounds	Interactions	Train	Test
Davis	442	68	30,056	25,046	5010
KIBA	229	2111	118,254	98,545	19,709

### Molecular encoder

For each drug molecule in the experimental dataset, it is represented by its corresponding SMILES. The open source cheminformatics software RDKit [34] is used to convert SMILES string into its corresponding molecular graph. For the node features, we use a set of atomic feature representations adopted from DeepChem [35]. In order to better explore the features of molecule, Graph Isomorphism Network (GIN) is used as the graph encoder in this paper. GIN provides better inductive bias for graph representation learning, which generates node representations by repeatedly aggregating information from the local neighborhood nodes. After each GIN layer, there is a batch normalization layer activated by the ReLU function. Specifically, GIN uses a MLP model to update the node features and its update process can be written as:

2 $$\begin{aligned} \textbf{x}_{i}^{l + 1} = {{MLP}\left( {\left( {1 + \varepsilon } \right) \textbf{x}_{i}^{l} + {\sum \limits _{j \in \mathcal {N}_{i}}\textbf{x}_{j}^{l}}} \right) } \end{aligned}$$

where $\varepsilon$ is either a learnable parameter or fixed scalar, $\textbf{x}_{i}^{l}$ denotes the node feature of the i-th node in the l-th layer, $\mathcal {N}_{i}$ are neighborhoods to node i, and $\textbf{x}_{j}^{l}$ denotes the node features of the j-th node in the l-th layer.

Multiple GIN layers could aggregate information of node from its multi-hop neighbors, and the information embedded in the representations of different hops will gradually change from local information to global information. After L layers of GIN, a list of node representations $\left\{ {\textbf{x}_{i}^{0},\textbf{x}_{i}^{1},\ldots ,\textbf{x}_{i}^{L}} \right\}$ is generated. To avoid loss of node information, a convolution kernel of size (L, 1) called $\textbf{Conv}$ is used to aggregate node representations at different layers as Eq. 3, thus local and global information can be combined.

3 $$\begin{aligned} x_{i}^{G} = \textbf{C}\textbf{o}\textbf{n}\textbf{v}\left( \left[{\textbf{x}_{i}^{0},\textbf{x}_{i}^{1},\ldots ,\textbf{x}_{i}^{L}} \right]\right) \end{aligned}$$

After obtaining the final node embeddings containing information at different levels of the graph, the obtained embeddings are aggregated into fixed-length graph-level representations using a read-out function. In this paper, we use a global summation pooling function which we called as $\textbf{GlobalAddPool}$ to read out the representation *h*(*G*) of the nodes as Eq. 4:

4 $$\begin{aligned} h(G) = \textbf{G}\textbf{l}\textbf{o}\textbf{b}\textbf{a}\textbf{l}\textbf{A}\textbf{d} \textbf{d}\textbf{P}\textbf{o}\textbf{o}\textbf{l}\left( X^{G} \right) \end{aligned}$$

where the ${X}^{G}$ represent the node feature matrix. It returns batched graph-level output by aggregating node features across the node dimension, thus ensuring that the global representation of graph is more comprehensive.

### Contrastive learning method for molecular representation

**Fig. 2:**
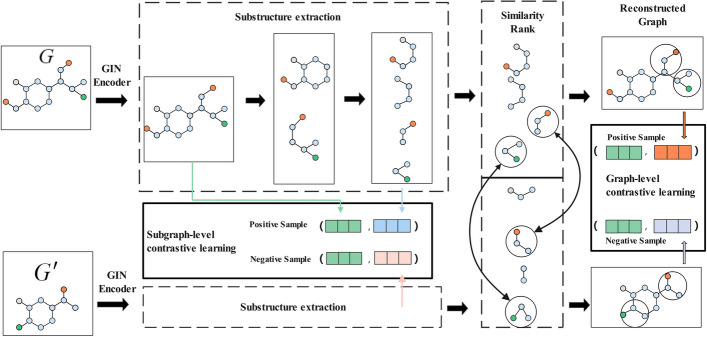
Contrastive learning method for molecular representation

Inspired by the mutual information-based contrastive learning algorithm [36, 37], maximizing the mutual information of molecular graphs can obtain more feature representations. The overall framework of our approach is shown in Fig. 2. The drug molecule graph acquires the original features after GIN encoding, followed by substructure extraction. For the original graph, its original feature is selected to form a positive sample pair with each subgraph representations, and the subgraphs of other graphs in the same batch form negative sample pairs. In order to capture the inherent relations between graphs, subgraphs are ranked according to similarity and half of them are replaced to obtain the reconstructed graph. The original graph with its reconstructed graph constitutes a positive sample, and with the reconstructed graph within the same batch constitutes a negative sample.

### Subgraph-level contrastive learning

In this paper, a subgraph’s generation method [29] is utilized in contrastive learning. After obtaining the node feature matrix $X^{G}$, it is transformed by linearly function with the learnable matrix *W* and the row-by-row $\textbf{Softmax}$ function is used to obtain a probability matrix *A* as Eq. 5. $A_{ij}$ denotes the probability of the i-th node in the j-th subgraph. The $\textbf{Softmax}$ function exponentiates the input vectors and sums them to obtain a scalar. The exponent value of each element is then divided by this scalar to obtain the normalized probability value.

5 $$\begin{aligned} A = \textbf{S}\textbf{o}\textbf{f}\textbf{t} \textbf{m}\textbf{a}\textbf{x}\left( {X^{G} \cdot W} \right) \end{aligned}$$

Based on the probability matrix *A*, we can divide the original graph into two subgraphs by a pre-defined probability 0.5. After *T* rounds of splitting, we obtain $S = 2^{T}$ subgraphs. The node representations of each subgraph are denoted as $X^{G_{i}},~i = 1,~2,~\ldots ,~S$. Here, we adopt the same pooling function as Eq. 4 to obtain the graph-level representation. After the reading out function, we can obtain the subgraph representation $h\left( G_{i} \right)$ as Eq. 6:

6 $$\begin{aligned} h\left( G_{i} \right) = \textbf{G}\textbf{l}\textbf{o}\textbf{b}\textbf{a}\textbf{l}\textbf{A} \textbf{d}\textbf{d}\textbf{P}\textbf{o}\textbf{o}\textbf{l}\left( X^{G_{i}} \right) ,i = 1,2,\ldots ,S \end{aligned}$$

Mutual information (MI) is an indicator to quantify the relationship between two random variables. Let $\phi$ represent the parameters of the graph neural network, and a discriminator $T_{\omega }:~h_{\phi }(G){\times h}_{\phi }\left( G_{i} \right)$ which takes as input a subgraph/graph embedding pair and determines whether they come from the same graph is used:

7 $$\begin{aligned} \overset{\hat{}}{\phi },\overset{\hat{}}{\omega } = \underset{\phi ,\omega }{\arg {max}}{\sum \limits _{G \in \textbf{G}}\frac{1}{\left| \textbf{G} \right| }}I_{\phi ,\omega } \left( {h_{\phi }(G);h_{\phi }\left( G_{i} \right) } \right) \end{aligned}$$

where $I_{\phi ,\omega }\left( h_{\phi }(G);h_{\phi }\left( G_{i} \right) \right.$ is a mutual information estimator modeled by the discriminator and parameterized by the neural network.

We use the Jensen–Shannon (JS) mutual information estimator [38] on local/global pairs to maximize the mutual information on a given subgraph/graph embedding as Eq. 8. The JS mutual information estimator is approximately monotonic with respect to the KL scatter (the traditional definition of mutual information), but it is more stable and can provide better results [39].

8 $$\begin{aligned} \begin{array}{l} I_{\phi ,\omega }^{JSD}\left( {h_{\phi }(G);h_{\phi }\left( G_{i} \right) } \right) = \\ \textbf{E}_{P}(-sp(-T_{\omega }(h_{\phi }(G), h_{\phi }( G_{i}^{})))) \mathbf {-} \textbf{E}_{P}(sp(T_{\omega }(h_{\phi }(G), h_{\phi }( G_{i}^{'})))) \\ \end{array} \end{aligned}$$

where $P = p\left( h_{\phi }(G),h_{\phi }\left( G_{i} \right) \right)$ is the joint distribution of the global graph representation and the subgraph representation, and $Q = p\left( h_{\phi }(G) \right) p\left( h_{\phi }\left( G_{i} \right) \right)$ denotes the product of marginal distributions of two embeddings. In contrastive learning, Q denotes the distribution of positive pairs, *P* denotes the distribution of negative pairs, and $sp(x) = log\left( 1 + e^{x} \right)$ is the softplus function.

### Graph-level contrastive learning

The reconstructed graph generation method is based on the strategy of similar subgraph substitution. To better capture the structural information of the graph, given the generated subgraph of a certain original graph $G_i$, we compute its cosine similarity to the generated subgraphs of other original graphs $G_i$ in the same batch as Eq. 9:

9 $$\begin{aligned} similarity = {\cos (\theta )} = \frac{h\left( G_{i} \right) \cdot h( G_{i}^{'} )}{\left\| {h\left( G_{i} \right) } \right\| \left\| {h( G_{i}^{'} )} \right\| } \end{aligned}$$

After ranking, half of the original subgraphs are replaced according to the similarity values, and finally aggregated and assembled into a reconstructed graph using a convolution kernel of size (*S*, 1).

For the reconstructed representation $h( \hat{G} )$, the global feature *h*(*G*) of its original graph is selected to form a positive sample pair, and the negative sample pair constitute the reconstructed graph $h( \hat{G}' )$ of other graphs in the same batch. We use the same mutual information calculation method to maximize the mutual information between the original and reconstructed graphs, denoted as $I_{\phi ,\omega }^{JSD}( {h_{\phi }(G);h_{\phi }( \hat{G} )} )$ as Eqs. 10 and 11:

10 $$\begin{aligned} \overset{\hat{}}{\phi },\overset{\hat{}}{\omega } = \underset{\phi ,\omega }{\arg {max}} {\sum \limits _{G \in \textbf{G}}\frac{1}{\left| \textbf{G} \right| }}I_{\phi ,\omega }( {h_{\phi }(G);h_{\phi }( \hat{G} )} ) \end{aligned}$$

11 $$\begin{aligned} \begin{array}{l} I_{\phi ,\omega }^{JSD}( {h_{\phi }(G);h_{\phi }( \hat{G} )} ) = \\ \textbf{E}_{P}(-sp(-T_{\omega }(h_{\phi }(G), h_{\phi }( \hat{G})))) \mathbf {-} \textbf{E}_{P}(sp(T_{\omega }(h_{\phi }(G), h_{\phi }( \hat{G}^{'})))) \\ \end{array} \end{aligned}$$

The final loss is the sum of two mutual information losses:

12 $$\begin{aligned} {Loss}_{\phi ,\omega } = I_{\phi ,\omega }^{JSD}\left( {h_{\phi }(G);h_{\phi }\left( G_{i} \right) } \right) + I_{\phi ,\omega }^{JSD}( {h_{\phi }(G);h_{\phi }( \hat{G} )}) \end{aligned}$$

To enhance the generalization of the self-supervised learning features, 50,000 molecules are randomly selected from the ZINC database for pre-training the self-supervised model, and a high-quality molecular encoder is obtained from learning rich molecular structure and semantic information in unlabeled molecular data.

### Protein representation

For each protein in the experimental dataset, the protein sequence is obtained from the UniProt database through its gene name. The sequence is a string of ASCII characters representing amino acids. The n-gram [40] is used to define the “words” in the amino acid sequence, and the protein sequence is split into multiple overlapping n-gram amino acid word. Depending on the permutations and combinations, there are $22^n$ n-gram words. However, if the n-gram syntax number is too large, the word frequency may be too low. Taking n = 3 as an example, given a protein sequence $S = s_{1}s_{2}s_{3}\ldots s_{|s|}$, |*S*| represents the length of protein sequence, we divide it into n-gram words:

13 $$\begin{aligned} \left[{s_{1};s_{2};s_{3}} \right],\left[{s_{2};s_{3};s_{4}} \right], \ldots ,\left[{s_{{|S|} - 2};s_{{|S|} - 1};s_{|S|}} \right] \end{aligned}$$

We use the symbol $s_{i:i + 2}$ to represent the protein word $\left[s_{i};s_{i + 1};s_{i + 2} \right]$, and then encode the word using the Eq. 14.

14 $$\begin{aligned} c_{i} = \textbf{E}\textbf{m}\textbf{b}\textbf{e}\textbf{d}\textbf{d} \textbf{i}\textbf{n}\textbf{g}\left( s_{i:i + 2} \right) \end{aligned}$$

where the $\textbf{Embedding}$ function initializes the weight from the standard normal distribution according to the input vocabulary size and embedding dimension, and outputs the word vector corresponding to the vocabulary index in the weight.

In this work, inspired by MGraphDTA [21], we set n = 2, 3, 4 to encode protein respectively in order to detect the local residue patterns of proteins at different scales. Finally we get three types of embedding $c_{i}^{2},c_{i}^{3},c_{i}^{4}$. For protein sequence, sequence-based models are the optimal choice for feature extraction. Long short-term memory network (LSTM) [41] is a DL model to overcome the gradient disappearance problem to process sequence data. The main idea is to introduce an adaptive gating mechanism which determines the extent to which the LSTM unit maintains its previous state and remembers the extracted features of the current data input.

Bi-directional LSTM (BiLSTM) [42] is a variant of LSTM that combines the outputs of two LSTMs, one processing sequences from left to right and the other from right to left, to capture long-term dependencies and contextual relationships. Since each amino acid residue in the sequence information of the protein has interrelationship with residues in the both directions, the BiLSTM is more suitable to process protein sequence, which is defined as Eq. 15:

15 $$\begin{aligned} \overset{\rightarrow }{h_{i}}&{= \overset{\rightarrow }{LSTM}\left( {c_{i},h_{i - 1}} \right) } \nonumber \\ \overset{\leftarrow }{h_{i}}&{= \overset{\leftarrow }{LSTM}\left( {c_{i},h_{i + 1}} \right) } \nonumber \\ h_{i}&{= \overset{\rightarrow }{h_{i}} \parallel \overset{\leftarrow }{h_{i}}} \end{aligned}$$

where $\overset{\rightarrow }{h_{i}}$ and $\overset{\leftarrow }{h_{i}}$ denote the hidden states of the time step computed from left-to-right and right-to-left, respectively, and $h_i$ denotes the global representation of the t-th time step stitched together by them.

**Fig. 3:**
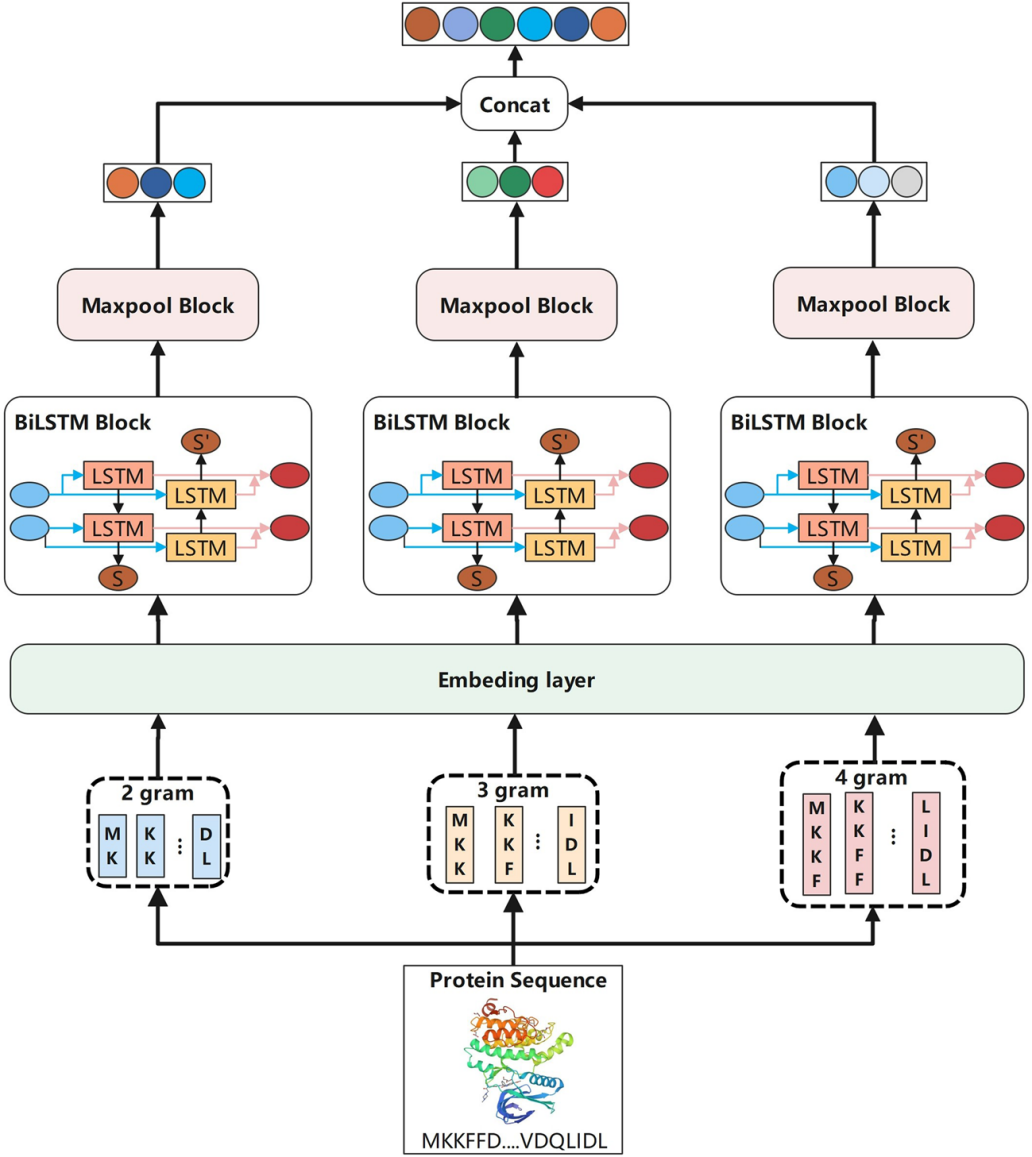
Multi-scale protein representation method

The word vector $c_{i}^{2},c_{i}^{3},c_{i}^{4}$ are fed into the BiLSTM layer to capture the dependencies between characters in the sequence. After the max-pooling layer, the three features are concatenated together to obtain the final protein representation. The BiLSTM framework is shown in Fig. 3.

### DTA prediction

**Fig. 4:**
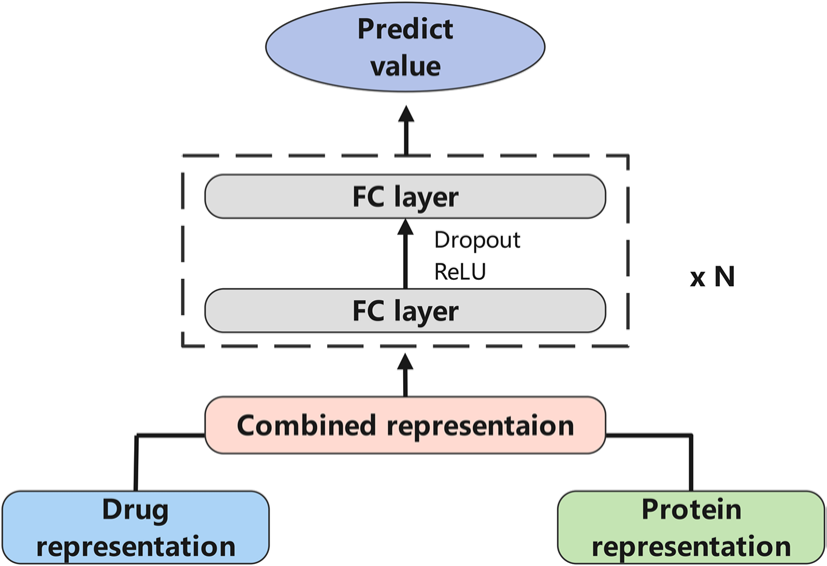
The prediction part of the model

In this paper, we treat the drug–target binding affinity prediction task as a regression task. With the representation learned from the previous sections, we can integrate all the information from the drug and target to predict the DTA value. As shown in Fig. 4, drug representation and protein representation are concatenated together, which is fed into two dense fully connected layers to predict the DTA value. Besides, the ReLU is used as the activation function for increasing the nonlinear relationship. Given the set of drug–target pairs and the ground-truth labels, we use the mean squared error (MSE) as the loss function.

## Results and discussion

### Metrics

The DTA prediction is regarded as a regression problem and our model was evaluated using three metrics including mean squared error (MSE), concordance index (CI), and regression toward the mean ($r_m^2$ index). MSE calculates difference between the predicted and actual values through the function of squared loss as follows:

16 $$\begin{aligned} MSE = \frac{1}{n}{\sum \limits _{i = 1}^{n}\left( {{\hat{y}}_{i} - y_{i}} \right) ^{2}} \end{aligned}$$

where ${\hat{y}}_{i}$ is the predicted value, $y_i$ is the true value, and n is the number of drug–target pairs. CI is used to measure whether the predicted DTA values of two random drug–target pairs are predicted in the same order as their true values:

17 $$\begin{aligned} CI= & {} \frac{1}{Z}{\sum \limits _{d_{x} > d_{y}}{h\left( {b_{x} - b_{y}} \right) }} \end{aligned}$$

18 $$\begin{aligned} h(x)= & {} \left\{ \begin{matrix} {1,~~~~~~~~if~x > 0} \\ {0.5,~~~~if~x = 0} \\ {0,~~~~~~~~if~x < 0} \\ \end{matrix} \right. \end{aligned}$$

where $b_x$ is the predicted value of the larger affinity $d_x$, $b_y$ is the predicted value of the smaller affinity $d_y$, *h*(*x*) is the step function. *Z* is the normalization constant which indicates the number of drug–target pairs.

$r_m^2$ is used to evaluate the external predictive performance of the model as follows:

19 $$\begin{aligned} r_{m}^{2} = r^{2} \times \left( {1 - \sqrt{r^{2} - r_{0}^{2}}} \right) \end{aligned}$$

where $r^2$ and $r_0^2$ are the squared correlation coefficients between the true and predicted values with and without intercepts, respectively.

### Comparison with existing methods

To evaluate the performance of our model, we compared the model with other methods for DTA prediction, including KronRLS [11], SimBoost [12], DeepDTA [16], WideDTA [43], MATT-DTI [26], DeepGS [44], AttentionDTA [45], GraphDTA [18], and DeepGLSTM [46]. Table 2 shows the performance of different models based on MSE, CI, and $r_m^2$ metrics on the Davis dataset. On the Davis dataset, our method significantly outperformed the other methods in terms of MSE (0.218) and $r_m^2$ (0.719), which are 4.8% and 4.8% better than the previous optimal method, respectively. The CI of SubMDTA was very close to the best method DeepGLSTM by 0.001.

**Table 2:** Prediction performance on Davis dataset

Model	MSE	CI	$r_m^2$
KronRLS	0.379	0.871	0.407
SimBoost	0.282	0.872	0.644
DeepDTA	0.261	0.878	0.630
WideDTA	0.262	0.886	0.633
MATT_DTI	0.229	0.890	0.682
DeepGS	0.252	0.882	0.686
AttentionDTA	0.245	0.887	0.657
GraphDTA	0.229	0.893	0.649
DeepGLSTM	0.232	0.895	0.680
SubMDTA	0.218	0.894	0.719

Moreover, we evaluated our model on KIBA dataset. As shown in Table 2, SubMDTA achieved the best performance among existing methods with MSE of 0.129, CI of 0.898, and $r_m^2$ of 0.793, where the MSE was 3% higher than the previous best method. The above results show that the proposed method can be considered as an accurate and effective tool for DTA prediction. Compared with other models, the superiority of our model can be summarized for two reasons: (i) to obtain more discriminative molecular representations, we utilized the local and global information of molecule through a pre-training task, which can focus on the structural features of molecular graph; (ii) compared with the conventional embedding method of protein sequence, our method used multiple n-gram sequence representations containing multi-level information. Thus, our model can integrate the intrinsic information of compounds and protein sequences into a more comprehensive representation, which is helpful to improve the accuracy and robustness of the model.

In addition, we evaluated our model on KIBA dataset. As shown in Table 3, SubMDTA achieved the best performance among existing methods with MSE of 0.129, CI of 0.898, and $r_m^2$ of 0.793, where the MSE was 3% higher than the previous best method.

**Table 3:** Prediction performance on KIBA dataset

Model	MSE	CI	$r_m^2$
KronRLS	0.411	0.782	0.342
SimBoost	0.222	0.836	0.629
DeepDTA	0.194	0.863	0.673
WideDTA	0.179	0.875	0.675
MATT_DTI	0.150	0.889	0.756
DeepGS	0.193	0.860	0.684
AttentionDTA	0.162	0.882	0.735
GraphDTA	0.147	0.889	0.674
DeepGLSTM	0.133	0.897	0.792
SubMDTA	0.129	0.898	0.793

The above results show that the proposed method can be considered as an accurate and effective tool for DTA prediction. Compared with other models, the superiority of our model can be summarized for two reasons: (i) to obtain more discriminative molecular representations, we utilized the local and global information of molecule through a pre-training task, which can focus on the structural features of molecular graph; (ii) compared with the conventional embedding method of protein sequence, our method used multiple n-gram sequence representations containing multi-level information. Thus, our model can integrate the intrinsic information of compounds and protein sequences into a more comprehensive representation, which is helpful to improve the accuracy and robustness of the model.

### Comparison with different drug molecular representations

**Fig. 5:**
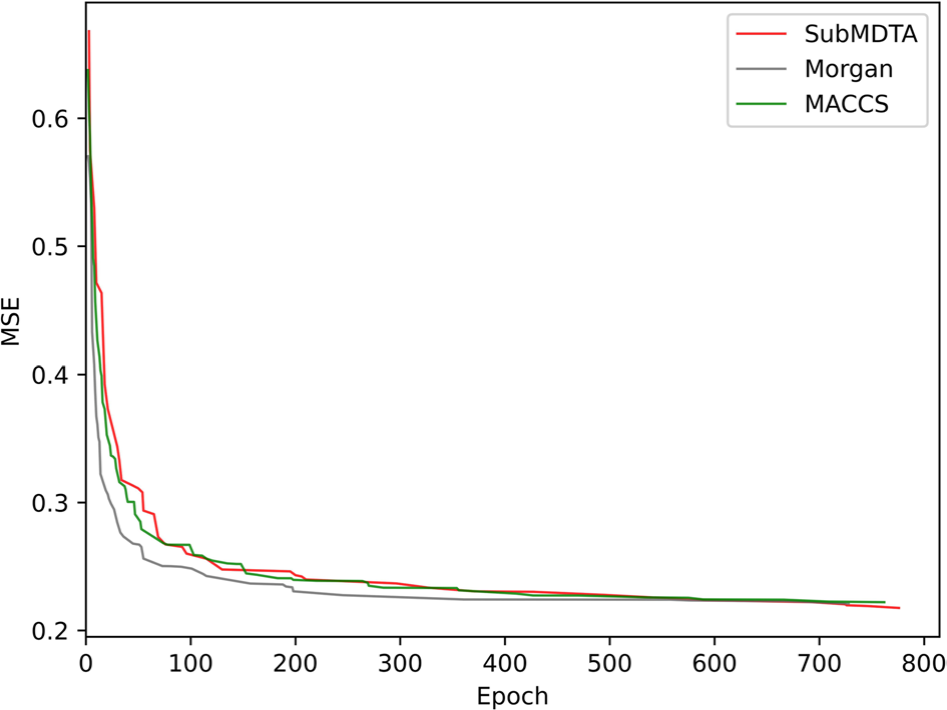
Performances of different molecular representation methods

The complex structure of drug molecules is difficult to directly obtain its features, so special representation methods are required. We validated graph-based representation methods and molecular fingerprint methods. SubMDTA first converts the smiles string of the drug molecule into a molecular graph, and then uses one-hot encoding to obtain the features of the drug molecule according to the atomic attributes. Molecular fingerprint is a method of converting a molecular structure into a binary or sparse vector representation, where each bit or feature represents a specific substructure or chemical property of the molecule. In this section, the Morgan fingerprint [47] and the MACCS fingerprint [48] were used for comparison. SubMDTA, Morgan, and MACCS achieved MSE of 0.218, 0.221, and 0.222, respectively. It can be seen from Fig. 5 that SubMDTA finally obtained the best results among three, which may be related to the fact that the graph-based method can better capture the detailed structure of molecules.

**Fig. 6:**
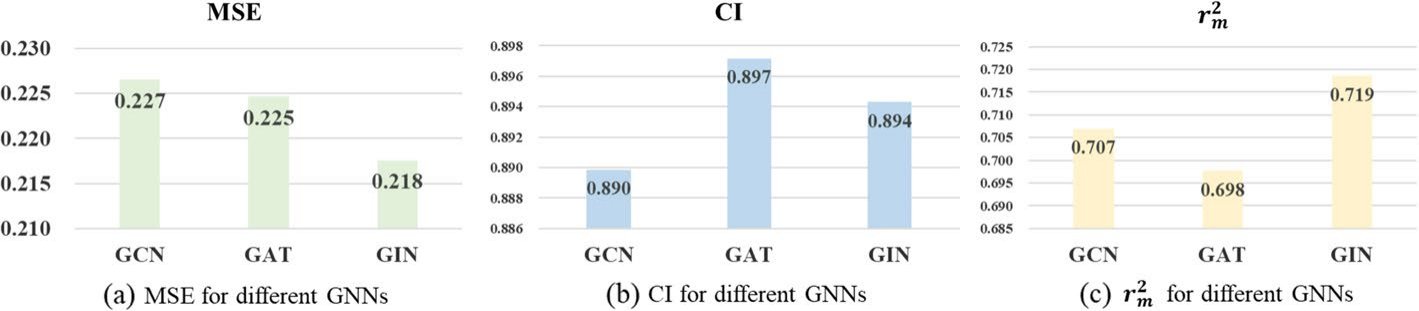
Performances of different GNNs

The construction of effective GNN networks for extracting discriminative features of drugs is essential to improve the prediction accuracy of DTA. Empirically, it is often difficult to obtain sufficient information from single-layer networks compared with multilayer networks, and too many layers may result in the problem of over-smoothing. Therefore, a four-layer GNN network was used in the proposed method. We tried three types of GNN architectures (GCN, GAT, and GIN) for performance comparison. It is obvious from the Fig. 6a and the Fig. 6c that the GIN model achieves an MSE of 0.218 and fran $r_m^2$ of 0.719, which is the best performance. As shown in Fig. 6b, the CI of the GAT model achieves 0.897, which is higher than 0.894 of GIN, but the difference is not obvious. This may be because that GIN can capture local features in the graph while retaining global information, thus improving its characterization ability.

### Comparison with different protein representations

**Fig. 7:**
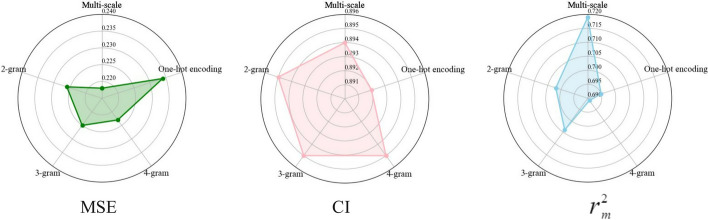
Performances of different protein feature extraction methods

For protein feature representation, we propose a method based on n-gram multi-scale features fusion. Thus, we explored the effects of different protein sequence embedding methods, which are one-hot coding, 2-gram, 3-gram, 4-gram coding and n-gram fusion coding methods, and the experimental results are shown in Fig. 7. One-hot coding achieved an MSE of 0.234, CI of 0.892, and $r_m^2$ of 0.695. Compared with one-hot encoding, n-gram encoding provided better representations by capturing multiple characters in the sequence, and the MSE reached 0.226, 0.225, and 0.223 using 2-gram, 3-gram, and 4-gram, respectively.

The performance of multi-scale representations was the best among them. This is because that the whole protein sequence contains many subsequences or structural domains, and the introduction of multi-scale features could capture more amino acid combinations and result in a better performance.

**Fig. 8:**
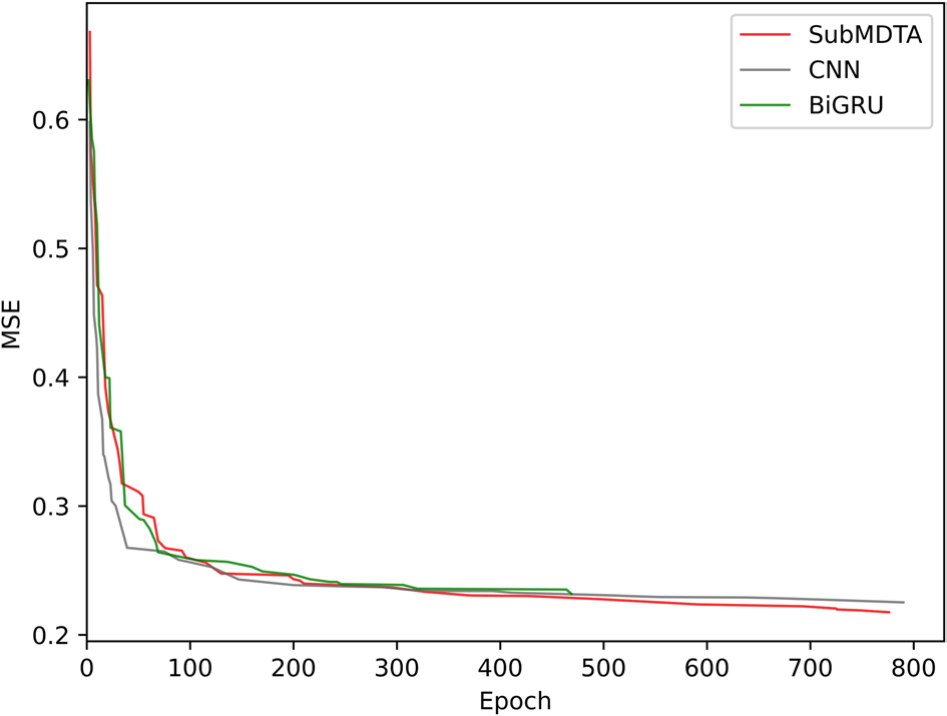
Performances of different protein feature extraction methods

For protein feature extraction methods, we choose convolutional neural network (CNN) and bidirectional gated recurrent unit (BiGRU) as comparison methods. CNN extracts features from input data through convolution operations. BiGRU is a variant of recurrent neural network which consists of two GRUs for forward and backward processing. CNN and BiGRU achieved MSE of 0.225, and 0.232, respectively. SubMDTA achieved MSE of 0.219, which increased by 3.1% and 6.0%. As can be seen from Fig. 8, SubMDTA obtained the best MSE result, which proves the superiority of SubMDTA in processing protein sequence data.

### Ablation study

To verify the effectiveness of the proposed model, we designed and conducted ablation experiments to determine the contributions of different factors of the model.

**Table 4:** Performances of different molecular training tasks

Model	MSE	CI	$r_m^2$
SubMDTA-a(no pretraining)	0.224	0.894	0.714
SubMDTA-b (only subgraph-level pretraining)	0.225	0.897	0.703
SubMDTA-c (only graph-level pretraining)	0.230	0.895	0.697
SubMDTA (both subgraph-level and graph-level pretraining)	0.218	0.894	0.719

In the proposed model, maximizing the mutual information between the graph and the subgraph representations in the SSL task is helpful to preserve substructure information. In order to demonstrate the advantages of substructures, we designed three variants SubMDTA-a, SubMDTA-b and SubMDTA-c to evaluate the importance of the pre-training task module. As shown in Table 4, SubMDTA-a obtained an MSE of 0.224. The introduction of contrastive learning improved the MSE to 0.225 and 0.230 by SubMDTA-b and SubMDTA-c, respectively. The MSE of SubMDTA which combined these two methods reached 0.218. This may be related to the fact that using one type of mutual information alone cannot obtain the comprehensive features. Meanwhile, maximizing the mutual information between the graph representation and the reconstructed graph representation can enable the embedding to focus on the global features of the graph.

### Case study

In order to verify the robustness of proposed method, we applied approved drugs targeting the Type-1 angiotensin II receptor in DrugBank for a case study. According to similar steps to MSF-DTA [49], after training SubMDTA on the Davis dataset, we predicted the affinities between the receptor and 1781 available small molecule drugs. Among them, 9 out of 1781 drugs are known to bind this receptor. To ensure a fair comparison, this receptor never appeared in the Davis dataset. The predicted affinities between the nine drugs and the receptor are listed in descending order, as shown in Table 5. It can be seen that according to the sorting results of SubMDTA, 8 drugs are ranked in the top 13 % of 1781 drugs, and 7 drugs appear in the top 4 %. These results suggest that SubMDTA can identify novel target-protein interacting drugs well and has the potential to be developed as a predictive tool.

**Table 5:** Compound ranking based on the predicted affinities of SubMDTA

DrugBank ID	Rank
DB00678	21
DB01029	24
DB00275	28
DB00966	30
DB00177	42
DB00796	51
DB08822	74
DB00876	224
DB11842	1130

## Conclusion

In this paper, we present a new model SubMDTA using self-supervised learning and multi-scale features for DTA prediction. The drug representations are extracted by contrastive learning methods between graph-level and subgraph representations and between graph-level and reconstructed graph representations, which is refined by downstream task. In addition, multi-scale sequence features were fused to learn protein representations, which captured long distance and multiple relationships in amino acid sequences. The experimental results proved that our method outperformed existing methods. In our future work, we will take account into the progresses made in heterogeneous information networks [50] and incorporate them to enhance the prediction ability of our models.

## Data Availability

The code and data are provided at https://github.com/1q84er/SubMDTA

## Abbreviations

DTA: Drug–target affinity
DL: Deep learning
SMILES: Simplified molecular input line entry system
BiLSTM: Bi-directional long short-term memory
LSTM: Long short-term memory
GCN: Graph convolutional networks
GAT: Graph attention networks
GIN: Graph isomorphism networks
SSL: Self-supervised learning
